# Enhancing Adoptive Cell Transfer with Combination BRAF-MEK and CDK4/6 Inhibitors in Melanoma

**DOI:** 10.3390/cancers13246342

**Published:** 2021-12-17

**Authors:** Peter Kar Han Lau, Carleen Cullinane, Susan Jackson, Rachael Walker, Lorey K. Smith, Alison Slater, Laura Kirby, Riyaben P. Patel, Bianca von Scheidt, Clare Y. Slaney, Grant A. McArthur, Karen E. Sheppard

**Affiliations:** 1 Research Division, Peter MacCallum Cancer Centre, Melbourne, VIC 3000, Australia; peter.lau@petermac.org (P.K.H.L.); carleen.cullinane@petermac.org (C.C.); susan.jackson@petermac.org (S.J.); rachael.walker@petermac.org (R.W.); lorey.smith@petermac.org (L.K.S.); alison.slater@petermac.org (A.S.); laura.kirby@petermac.org (L.K.); Riyaben.patel@petermac.org (R.P.P.); bianca.vonscheidt@petermac.org (B.v.S.); clare.slaney@petermac.org (C.Y.S.); grant.mcarthur@petermac.org (G.A.M.); 2 Department of Medical Oncology, Peter MacCallum Cancer Centre, Melbourne, VIC 3000, Australia; 3 Sir Peter MacCallum Department of Oncology, University of Melbourne, Parkville, VIC 3010, Australia; 4 Department of Biochemistry and Pharmacology, University of Melbourne, Melbourne, VIC 3010, Australia

**Keywords:** melanoma, adoptive cell transfer, targeted therapy, cell cycle, immuno-oncology

## Abstract

**Simple Summary:**

Adoptive cell transfer (ACT) is a potentially robust treatment option for patients with advanced melanoma that is resistant to immune checkpoint inhibitors. The addition of cyclin-dependent kinase 4/6 inhibitors to combination BRAF-MEK inhibitors can also greatly improve the duration of response against melanoma. The aim of our study was to investigate adoptive cell transfer with combination BRAF-MEK and CDK4/6 inhibitors. We show triplet targeted therapy is highly efficacious against BRAF^V600^ melanoma in YOVAL1.1 and the BRAFi resistant SM1WT1 model. Combination ACT with BRAF-MEK-CDK4/6i led to prolonged and deep anti-tumor responses in YOVAL1.1. This work provides additional evidence for BRAF-MEK-CDK4/6i in clinical trials and in combination with ACT.

**Abstract:**

Despite the success of immune checkpoint inhibitors that target cytotoxic lymphocyte antigen-4 (CTLA-4) and programmed-cell-death-1 (PD-1) in the treatment of metastatic melanoma, there is still great need to develop robust options for patients who are refractory to first line immunotherapy. As such there has been a resurgence in interest of adoptive cell transfer (ACT) particularly derived from tumor infiltrating lymphocytes. Moreover, the addition of cyclin dependent kinase 4/6 inhibitors (CDK4/6i) have been shown to greatly extend duration of response in combination with BRAF-MEK inhibitors (BRAF-MEKi) in pre-clinical models of melanoma. We therefore investigated whether combinations of BRAF-MEK-CDK4/6i and ACT were efficacious in murine models of melanoma. Triplet targeted therapy of BRAF-MEK-CDK4/6i with OT-1 ACT led to sustained and robust anti-tumor responses in BRAFi sensitive YOVAL1.1. We also show that BRAF-MEKi but not CDK4/6i enhanced MHC Class I expression in melanoma cell lines in vitro. Paradoxically CDK4/6i in low concentrations of IFN-γ reduced expression of MHC Class I and PD-L1 in YOVAL1.1. Overall, this work provides additional pre-clinical evidence to pursue combination of BRAF-MEK-CDK4/6i and to combine this combination with ACT in the clinic.

## 1. Introduction

While immune checkpoint inhibitors (ICI) targeting cytotoxic lymphocyte antigen-4 (CTLA-4) and anti-programmed death-1 (anti-PD1) have taken center stage in the treatment of advanced melanoma, there is a paucity of robust systemic treatment options in the refractory setting. Combination ipilimumab-nivolumab is generally favored as first line treatment for BRAF^V600^ metastatic melanoma given its five-year progression free survival (PFS) rate is double that of upfront BRAF-MEK inhibitors such as dabrafenib-trametinib (38% vs. 19%) [1,2]. As such, targeted therapy with BRAF-MEKi is increasingly used as second line therapy after immune checkpoint inhibitors but has relatively short duration of response of around 11–12 months [2]. Adoptive cell transfer (ACT) derived from tumor infiltrating lymphocytes (TIL) is a potentially robust option in the ICI refractory setting. Original studies by Rosenberg and colleagues, showed an objective response rate of approximately 50% with almost 30% of patients alive at the five-year timepoint in a pooled analysis of 3 trials [3,4]. Owing to the high labor requirements of extraction and production, TIL ACT has been limited to highly specialized academic centers. However, lifileucel is a commercial, centrally manufactured TIL ACT product that has shown promising activity in pre-treated ICI patients (*n* = 66) with an objective response of 36% [5]. Although median PFS was 4.1 months, the median duration of response was not reached after 18 months indicating the potential of long-term responses.

Combining BRAF-MEKi with ACT could therefore harness the advantages of targeted therapy with its high response rate (60–70%) and immunotherapy with its capacity for long term disease control. Findings from earlier pre-clinical studies support this approach. The addition of BRAFi (vemurafenib) to pmel-1 and OT-1 ACT treatments enhanced anti-tumor responses against the BRAFi resistant murine melanoma SM1 and SM1-ova tumors, respectively [6]. In a subsequent study, Hu-Lieskovan et al. [7], demonstrated synergy of combination BRAF-MEKi (dabrafenib and trametinib) with pmel-1 ACT against SM1 tumors. By itself, pmel-1 ACT had modest activity, but once combined with BRAF-MEKi, substantial tumor control was observed. Although anti-tumor responses to BRAF-MEKi are largely attributable to the sequential blockade of the mitogen activated protein kinase (MAPK) pathway, pre-clinical and patient studies show targeted therapy can exert a range of immunomodulatory effects on melanoma which are potentially synergistic with ACT [8]. BRAFi upregulates melanoma differentiation antigens such as gp100, MART-1 and tyrosinase in murine models [7], in vitro human cell lines [9,10], and in patient biopsy samples [11]. Additionally, expression of MHC I expression can be upregulated by melanoma in response to BRAFi treatment [10]. Hence targeted therapy may enhance the immunogenicity of melanoma and potentially augment the activity of ACT.

Cyclin dependent kinase 4/6 inhibitors (CDK4/6i) may also play a role in enhancing duration of response in combination with BRAF-MEKi via blockade of the cell cycle and exerting immunomodulatory effects on melanoma. The addition of CDK4/6i such as palbociclib [12] or ribociclib [13] to endocrine therapy almost doubles progression free survival compared to aromatase inhibitors alone in metastatic oestrogen receptor positive breast cancer. Approximately 90% of melanoma exhibit some cell cycle pathway dysregulation [14] which provides a strong rationale to combine CDK4/6i with BRAF-MEKi. Studies in our laboratory have shown encouraging in vivo anti-melanoma activity of palbociclib in combination with BRAF-MEKi [15,16]. Additional pre-clinical studies also demonstrate potential immuno-modulation by CDK4/6 inhibitors (CDK4/6i) in breast cancer and melanoma with upregulation of MHC Class I expression, T regulatory cell reduction, increased IFN-γ release in the tumor microenvironment and enhanced long-term anti-tumor immunity [17,18,19,20,21].

Building upon previous studies showing synergistic activity of BRAF-MEKi with ACT, we investigated whether the addition of CDK4/6i could further improve anti-tumor responses. We use two pre-clinical models, YOVAL1.1 which expresses the ovalbumin (ova) antigen and SM1WT1 which expresses gp100 which are amenable to ACT such as OT-1 and pmel-1, respectively. The SM1WT1 cell line is derived from SM1 which is resistant to BRAF inhibitors by nature of a BRAF amplification and transversion thereby providing a suitable platform to study the response in the BRAFi resistance setting [6,22]. Whereas YOVAL1.1 harbours a BRAF^V600E^ mutation that is susceptible to BRAFi treatment. Importantly both YOVAL1.1 and SM1WT1 exhibit a *CDKN2A* deletion which encodes the tumor suppressor p16 which is an inhibitor of CDK4/6 and thus the cell cycle pathway. Hence both cell lines provide a mechanistic rationale for the addition of CDK4/6 inhibitor in melanoma.

## 2. Materials and Methods

### 2.1. Cell Culture and Drugs

The SM1WT1 cell line was derived at Peter MacCallum Cancer Centre from the SM1 cell line [22]. The YOVAL1.1 cell line was derived in our laboratory from YUMM1.1 [23]. SM1WT1 was maintained in Roswell Park Memorial Institute (RPMI) media supplemented with 10% (*v*/*v*) fetal bovine serum (FBS) and Glutamax (35050-061, Life Technologies, Carlsbad, CA, USA). YOVAL1.1 cells were maintained in RPMI supplemented with 10% (*v*/*v*) fetal bovine serum (FBS), glutamax, 1 mM sodium pyruvate, 1 mM MEM Non-Essential Amino Acids, and 0.1% 2-mercaptoethanol. Dabrafenib and trametinib were purchased from Ark Pharm (catalog No. AK174048, Wuhan, Hubei, China) and Focus Bioscience (catalog No. HY-10999, Queensland, Australia), respectively, and palbociclib was from Pfizer Oncology (New York, NY, USA).

### 2.2. Cell Proliferation Assays

Cell proliferation assays were performed as previously described [15]. Plates were imaged using the Incucyte, with continuous live cell imaging (Essen Bioscience, Ann Arbor, MI, USA) every 12 h. Cell confluence was calculated as a marker of proliferation from 4 fields per well.

### 2.3. Gene Expression Analysis

Total RNA was isolated using the RNeasy Mini kit (Qiagen, Hilden, Germany) following the manufacturer’s instructions. Purity and concentration of RNA was assessed using the Nanodrop ND1000 spectrophotometer (Thermo Scientific, Waltam, MA, USA). Complementary DNA (cDNA) was generated using the High Capacity cDNA Reverse Transcription Kit (Life Technologies). Quantitative real-time PCR using Mastermix Lightcycler SBR Green Master Mix (Roche, Basel, Switzerland) was performed on a Lightcycler 480 (Roche). NONO RNA was plotted as relative expression values. H2K1 (Biorad PrimePCR Assay H2K1, Catalog 10025636), and TAP1 (Biorad PrimePCR Assay Tap1, Catalog 10025636) were sourced from Biorad (Richmond, CA, USA). The primer sequences are as follows:

B2M-Forward: TTCACCCCCACTGAG
B2M-Reverse: GTCTTGGGCTCGGCC
NONO-Forward: TATCAGGGGGAAGATTGCCC
NONO-Reverse: GCCAGAATGAAGGCTTGACT

### 2.4. Animal Work

All in vivo mice experiments were approved by the Animal Ethics Committee of Peter MacCallum Cancer Centre (Protocol E569). Male C57Bl/6 mice aged between 5 and 6 weeks were purchased from Walter and Eliza Hall Institute (Melbourne, Australia). Mice were housed in pathogen free conditions at the Peter MacCallum Cancer Centre Animal Hold facility.

Briefly mice were shaved on the right flank the day prior to implantation and were anaesthetized using inhaled isoflurane and then injected with freshly harvested 1 × 10^6^ tumor cells in 0.1 mL of sterile PBS. For the SM1WT1 model only, male C57/Bl6 mice aged 12–16 weeks of age were exposed to 4 Gy radiation using the XRAD 320 irradiator immediately prior to tumor establishment. Tumor measurements commenced 5 days after implantation and were performed two or three times weekly using digital calipers. Tumor volume was calculated by the formula 0.5 × length × width squared. Mice were sacrificed when tumor volume reached 1200 mm^3^.

Dabrafenib-trametinib was prepared daily for administration in an aqueous solution of 0.5% hydroxypropyl methylcellulose and 0.2% Tween 80 (Sigma-Aldrich, St. Louis, MO, USA). Palbociclib was suspended in sodium lactate 50 mM. All mice were weighed prior to oral gavage. Dabrafenib and trametinib was administered at 30 mg/kg and 0.3 mg/kg, respectively, once daily. Palbociclib was dosed at 80 mg/kg once daily.

### 2.5. T Cell Expansion and Adoptive Cell Transfer

Male pmel-1 C57/Bl6 were euthanized and spleens dissected and placed into cold phosphate buffered saline (PBS) [24]. Spleens were minced and then incubated with ammonium-chloride-potassium lysis buffer for 1 min. Splenocytes were washed and filtered through a 70 micron filter twice prior to a manual cell count. Cell suspended to a concentration of 1 million cells/mL and supplemented with gp100 peptide, IL-2, and IL-7 at final concentrations of 0.1 μg/mL, 50 IU/mL, 0.2 ng/mL, respectively. Splenocytes were then incubated at 37 °C 5% CO_2_. Cells were inspected every 2 or 3 days and split in a 1:1 ratio and supplemented with IL-2 at final concentration of 50 IU/mL. The pmel-1 cells were injected via tail vein on day 6 of cell culture. IL-2 (5 × 10^5^ IU) was injected intraperitoneally twice daily for 3 days commencing on the day of pmel-1 injection.

Male OT-1 mice were subjected to the same red cell lysis and washing procedures as above, however each spleen was placed into 100 mL of media supplemented with sinfekel, IL-2, and IL-7 at final concentrations of (1 μL per 50 mL), 100 IU/mL, and 0.2 ng/mL, respectively, incubated at 37 °C in 5% CO_2_. Three days later the splenocyte cultures were counted then resuspended to a concentration of 4 × 10^6^ cells/mL in fresh media replenished with IL-2 and IL-7. Cells were incubated for a further 3 days until tail vein injection into the recipient mice.

### 2.6. Flow Cytometry

Samples were blocked with 2% normal mouse serum. Fixable yellow (L34959, Invitrogen, Carlsbad, CA, USA) or 7AAD (Cat 00699350, eBioscience, Waltham, MA, USA) was used to stain live/dead cells. Anti-mouse antibodies used were CD3 (17A2, eBioscience), CD4 (GK1.5, BioLegend, San Diego, CA, USA), TCR Vα2 (KB5-C20, BD Pharmingen), CD8a (53-6.7, BioLegend), CD44 (IM7, Biolegend), CD62L (MEL-14, eBioscience), H-2Db (KH95, BD Pharmingen), H-2Kb (AF6–88.5, BD Pharmingen), PD-L1 (MIH5, eBioscience). Anti-human antibodies used were HLA ABC (W6/32, Biolegend), PD-L1 (29E.283, Biolegend), anti-mouse IgG2a K Isotype (Biolegend), and anti-mouse IgG2b K (Biolegend).

Fluorescence was measured on BD LSR Fortessa X-20 or BD FACSVerse flow cytometer (BD Biosciences, North Ryde, NSW, Australia) and data analyzed using the FlowJo, LLC software (BD Biosciences, Franklin Lakes, NJ, USA).

### 2.7. IFN-γ Release Assay

SM1WT1 or SM1 were co-cultured with IFN-γ (2 ng/mK) or control for 24 h. SM1WT1 cells were then trypsinized and washed twice prior to pulsing with gp100 peptide (1 ng/mL) for 30 min and then washed twice prior to incubation with pmel-1 cells for 4 h. Supernatants were transferred to a second 96 well plate and frozen at −20 °C until IFN-γ enzyme-linked-immunosorbant assay (ELISA) was performed as previously described [25].

### 2.8. Chromium Release Assay

Target cells were labelled with 250 μCi/mL Chromium-51 (51Cr; Perkin Elmer, VIC Australia) for 45 min prior to culturing with effector cells at 37 °C. Supernatant was collected and 51Cr was measured by a gamma counter (Wallac Wizard). 51Cr release due to effector-mediated killing was calculated as %Release = [(51CrSAMPLE − 51CrSPONT)/(51CrTOTAL − 51CrSPONT)] × 100; where 51CrSPONT is spontaneous 51Cr release from target cells cultured without effector cells, and 51CrTOTAL is total chromium release from cells lysed with 10% Triton X-100.

### 2.9. Statistical Analysis

All statistical analyses were performed with the GraphPad Prism version 8 software (GraphPad Software Inc, La Jolla, CA, USA). As indicated comparison between two groups were analyzed using Student’s *t*-test. Multiple comparisons were analyzed using one-way analysis of variance (ANOVA). A *p*-value of <0.05 was considered significant.

## 3. Results

### 3.1. YOVAL1.1 but Not SM1WT1 Mouse Melanoma Cells Are Highly Sensitive to Combination BRAF-MEKi and the Addition of Palbociclib Enhances Inhibition of SM1WT1 Proliferation

**Figure 1:**
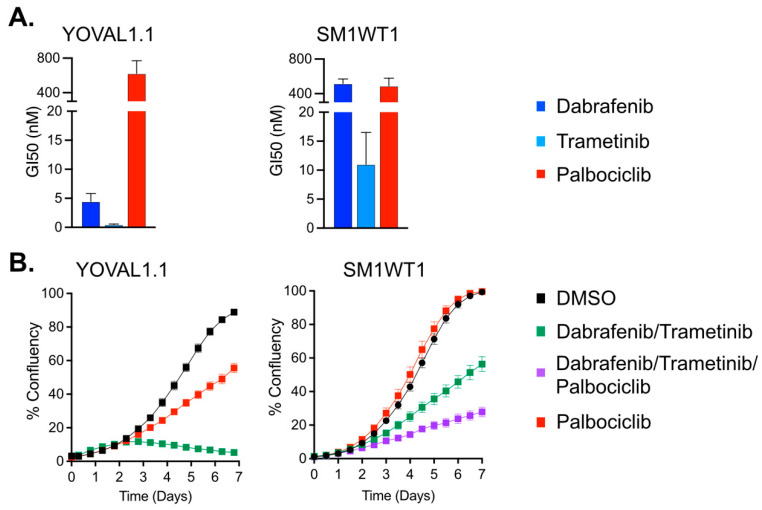
Sensitivity of mouse melanoma cell lines to targeted therapy. (**A**) Dose response assays were performed, and the concentration of drug required to inhibit proliferation by 50% (GI50) was determined. Bar graph represents mean ± SEM of 3–7 independent dose response experiments. For YOVAL1.1, a significant difference (*p* < 0.0001) in GI50′s was observed between palbociclib (623 ± 147 nM) and both dabrafenib (4.5 ± 1.3 nM) and trametinb (0.5 ± 0.1 nM). For SM1WT1, a significant difference (*p* < 0.0002) in GI50′s was observed between the trametinib (11.0 ± 3.2 nM) and both dabrafenib (519.0 ± 29.4 nM) and palbociclib (490 ± 52.0 nM). There was no difference in mean GI50 between the other groups within a cell line. One-way ANOVA was used to determine statistical significance. (**B**) To assess cell proliferation, cells were treated for 7 days, and percent confluency was analyzed over time using an IncuCyte. For YOVAL1.1, cells doses for dabrafenib, trametinib and palbociclib were 10 nM, 1 nM, and 2 µM, respectively. For SM1WT1, doses were 1 µM, 30 nM, and 1 µM, respectively. Mean ± SEM of 5 technical replicates.

We assessed the sensitivity of the YOVAL1.1 and SM1WT1 cell lines to dabrafenib (BRAFi), trametinib (MEKi), and palbociclib (CDK4/6i) (Figure 1A). YOVAL1.1 harbors a BRAF^V600E^ mutation, while in contrast SM1WT1 possesses a BRAF amplification rendering it resistant to BRAFi. YOVAL1.1 cells were sensitive to dabrafenib, trametinib, and moderately sensitive to palbociclib having GI50′s (dose required to inhibit growth by 50%) of 4.5 ± 1.3 nM, 0.5 ± 0.1 nM, and 623 ± 147 nM, respectively. In comparison, SM1WT1 cells were relatively resistant to both dabrafenib and trametinib but moderately sensitive to palbociclib, exhibiting GI50’s of 519 ± 29.4 nM, 11 ± 3.2 nM, and 490 ± 52 nM, respectively. To assess the activity of BRAFi, MEKi, and CDK4/6i combination therapy, cell proliferation over time was assessed (Figure 1B). Combination dabrafenib and trametinib (DT) at relatively low concentrations (10 nM and 1 nM, respectively) robustly inhibited cell proliferation in YOVAL1.1 cells, and palbociclib alone partially inhibited proliferation. In contrast, SM1WT1 cells exhibited resistance to both dabrafenib and trametinib at very high concentrations of 1 µM and 30 nM, respectively which only partially inhibited cell proliferation. However, the addition of palbociclib to DT elicited a more robust response.

### 3.2. Combination BRAF-MEK-CDK4/6i ± OT1 Is Highly Efficacious against YOVAL1.1

**Figure 2:**
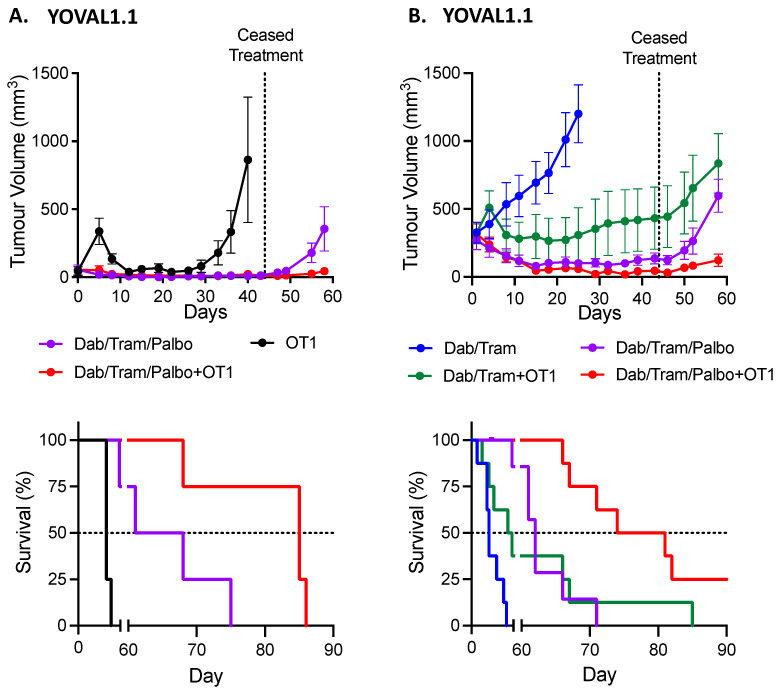
YOVAL1.1 tumor response to targeted and ACT. (**A**) In vivo YOVAL1.1 tumor volume growth and Kaplan–Meier overall survival curves of dabrafenib-trametinib-palbociclib (DTP ± OT-1 ACT with 4 mice per group (mean ± SEM). Gavages of DT ceased at day 44. Median overall survival (OS) for OT-1, DTP and DTP + OT-1 was 40, 64.5, and 85 days, respectively. OS hazard ratio (HR) for OT-1 versus DTP was 0.270, *p* = 0.006. OS HR of DTP versus DTP + OT-1 was 0.300, *p* = 0.03. (**B**) In vivo YOVAL1.1 tumor volume growth curve and Kaplan–Meier overall survival curves of dabrafenib-trametinib ± OT-1 ACT or dabrafenib-trametinib-palbociclib ± OT-1 ACT (mean ± SEM) with groups of 8 mice per each arm. Gavages of DT ceased at day 46. Median OS for DT, DTP, DT + OT-1 and DTP + OT-1 was 25, 62, 55, and 77.5 days, respectively. OS HR for DT versus DTP was 0.340, *p* = 0.02. OS HR for DTP versus DT + OT-1 was 1.154, *p* = 0.76. OS HR for DTP versus DTP + OT-1 was 0.250, *p* = 0.001. Hazard ratios determined with log-rank (Mantel-Cox).

Earlier in vivo studies in our laboratory have shown the triplet combination of BRAF-MEK-CDK4/6i leads to robust responses in YOVAL1.1 tumors that far exceed the response to combination of BRAF-MEKi [16]. We therefore assessed the impact of the addition of CDK4/6 inhibitor (palbociclib) in the BRAFi sensitive YOVAL1.1 cell line in conjunction with OT-1 ACT (Figure 2A). Syngeneic C57BL/6 mice were inoculated with YOVAL1.1 subcutaneously. When tumors were at a target size of 50 mm^3^, 5 × 10^6^ OT-1 cells were injected via tail vein and daily gavages of dabrafenib-trametinib-palbociclib (DTP) commenced. Administration of OT-1 cells alone led to an anti-tumor response; however, by day 26 tumors re-emerged. The triplet combination of DTP led to a robust and sustained YOVAL1.1 tumor regression compared to OT-1 cells alone. When OT-1 ACT was combined with DTP a similar robust and sustained response was observed. Given some tumors in both DTP arms were impalpable, oral gavages of the targeted therapy were ceased at day 44, which led to subsequent regrowth of all tumors. However, a significant difference (*p* < 0.03) in median survival was demonstrated between DTP (65 days) and DTP plus OT-1 (85 days).

In a second experiment (Figure 2B), we compared dabrafenib-trametinib (DT) or DTP with OT-1 for treating large tumors. DT alone did not induce tumor regression with median survival of 25 days. In contrast, DT + OT-1, DTP, DTP + OT1, and all led to strong anti-tumor responses that plateaued at day 15 in all groups, with mean volumes of 298 ± 163.0, 90 ± 28, and 46 ± 16 mm^3^ respectively. Again, due to the deep regressions observed with DTP, oral gavages of DT and DTP were ceased at day 46 and all but one tumor in the OT1 + DTP group displayed regrowth. Median survival of DTP + OT1 was 78 days which was superior to DT + OT-1 (55 days, *p* < 0.02). DTP was highly efficacious and superior to both DT alone and DT + OT-1. Similar to the previous experiment, there was significant additional anti-tumor activity, when OT-1 was combined with DTP compared to DTP alone (median survival of 77.5 days versus 62 days, *p* < 0.001). Between both experiments the DTP arms led to average weight loss of between 2.3% to 4.3% in the first 15 days of treatment which then returned to their mean baseline between day 23 and 46.

### 3.3. In the Therapy Resistant SM1WT1 Model, ACT Does Not Enhance Response to Combination BRAF-MEKi ± CDK4/6i

**Figure 3:**
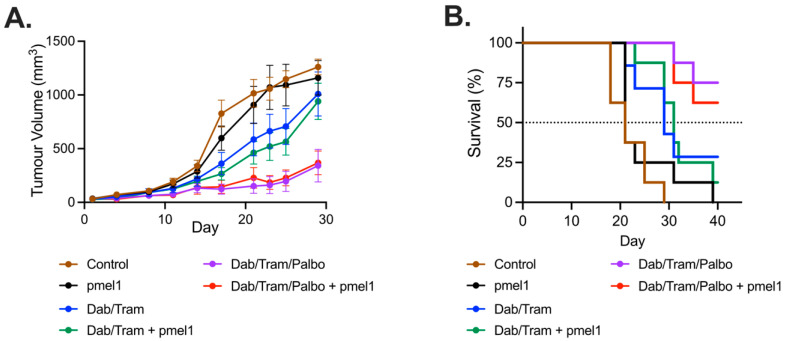
SM1WT1 tumor response to targeted therapy and ACT. (**A**) SM1WT1 tumor volume growth curves of dabrafenib-trametinib (DT) ± palbociclib (P) and pmel-1 adoptive cell transfer (ACT) with 8 mice per group (mean ± SEM). (**B**) Kaplan–Meier overall survival (OS) curves of data shown in (**A**). Median OS for control, pmel-1, DT, DT + pmel-1, DTP, and DTP + pmel-1 was 21, 21, 29, 31, not attained, and not attained, respectively. OS hazard ratio (HR) for control versus pmel-1 was 1.57, *p* = 0.32. OS HR for DT versus DT + pmel-1 was 0.995, *p* > 0.99. OS HR for DTP versus DTP + pmel-1 was 0.627, *p* = 0.58. OS HR for DT versus DTP was 4.695, *p* = 0.03. Hazard ratios determined with log-rank (Mantel-Cox).

Given the YOVAL1.1 in vivo results, we then sought to assess whether the addition of palbociclib to combination BRAF-MEKi and pmel-1 ACT would further enhance anti-tumor activity against the BRAFi resistant SM1WT1 tumors. Syngeneic C57BL/6 mice were subjected to 4 Gy radiation and then immediately inoculated with SM1WT1 subcutaneously. When tumors were at least 50 mm^3^, the mice received 1 × 10^7^ pmel-1 cells via tail vein injection or mock ACT both with 5 × 10^5^ IU of IL-2 intraperitoneal injection (control). Dabrafenib-trametinib ± palbociclib gavages were also commenced. There was no difference in the tumor growth curves between the control and pmel-1 ACT arms (Figure 3). The two DT containing arms exhibited slower growth compared to control and this was enhanced by the addition of palbociclib. Surprisingly, pmel-1 ACT did not enhance either DT or DTP responses. Median survival (Figure 2) was improved substantially with both DT and DTP compared to control (*p* < 0.01 and *p* < 0.001, respectively) and DTP improved survival compared to DT (*p* < 0.03).

### 3.4. Pmel-1 T Cells Kills SM1WT1 In Vitro Only with gp100 Stimulation

**Figure 4:**
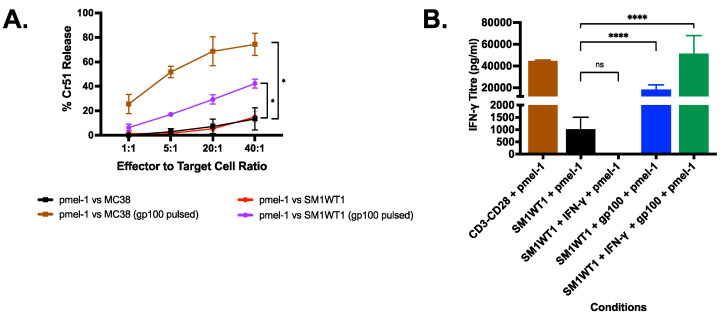
SM1WT1 tumor response to targeted and ACT. (**A**) Chromium release assay of MC38 and SM1WT1 vs. pmel-1 (mean ± SEM of 3 independent experiments). (**B**) SM1WT1-pmel-1 IFN-γ Release Assay (mean ± SEM of 3 independent experiments). SM1WT1 + pmel-1 were incubated for 16 h. To ensure MHC Class I expression, SM1WT1 cells were also pre-incubated with IFN-γ (2 ng/mL) for 24 h and washed twice prior to incubation with pmel-1 (SM1WT1 + IFN-γ + pmel-1). SM1WT1 was pulsed with gp100 peptide (1 ng/mL) for 30 min and washed twice prior to incubation with pmel-1 (SM1WT1 + gp100 + pmel-1). * *p* < 0.05, **** *p* < 0.0001.

To investigate the lack of response in the SM1WT1-pmel-1 system, pmel ACT cells were further assessed. Flow cytometry of the injected pmel-1 ACT cells showed a highly activated phenotype (Figure S1) with a high proportion of CD3, CD8, CD44^hi^, and CD62L^hi^ positive cells indicating a central memory phenotype. Chromium release assays were also performed to determine if pmel cells were effective at recognizing and killing SM1WT1 cells. Pmel-1 cells were able to kill approximately 80% of the highly immunogenic MC38 cells pulsed with gp100 and 40% SM1WT1 pulsed with gp100 with an effector:target ratio of 40:1 (Figure 4A). IFN-γ release assays (Figure 4B) showed low IFN-γ concentrations with pmel-1 and SM1WT co-cultures compared to pmel-1 and SM1WT cells pulsed with the gp100 peptide. These findings are surprising given we originally considered the melanoma SM1WT1 cells to express the classic gp100 melanoma peptide. To determine if SM1WT1 cells have a low expression of gp100 by MHC Class I, SM1WT1 cells were pre-incubated with IFN-γ for 24 h, to increase MHC Class I expression. Cells were washed extensively and then incubated with pmel-1 cells. Only upon pulsing IFN-γ stimulated SM1WT1 with gp100 peptide were high IFN-γ titers achieved; indicating low expression of this antigen as the likely cause for the poor activity of pmel-1 cells against SM1WT1 in vitro and potentially in vivo.

### 3.5. BRAF-MEKi Upregulates MHC Class I and PD-L1 in SM1WT1 and YOVAL1.1 In Vitro

**Figure 5:**
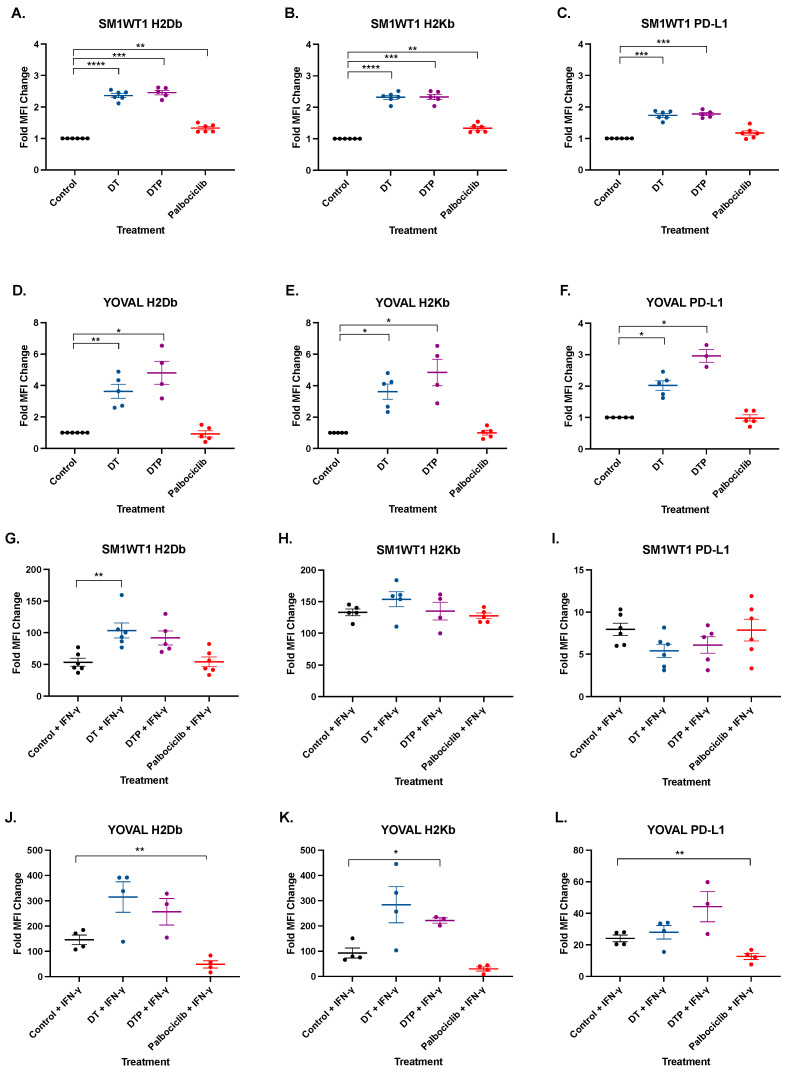
SM1WT1 and YOVAL1.1 MHC Class I and PD-L1 Expression. Samples analyzed using FACS for H2Db (**A**,**D**,**G**,**J**), H2Kb (**B**,**E**,**H**,**K**), and PD-L1 (**C**,**F**,**I**,**L**). SM1WT1 and YOVAL1.1 were treated with dabrafenib-trametinib (DT), dabrafenib-trametinib-palbociclib (DTP), or palbociclib alone. Median fluorescent intensity of the respective treatment group was normalized back to controls and expressed as fold MFI change. Mean ± SEM. *n* = 3–5. Statistical significance was determined using a one-way ANOVA * *p* < 0.05, ** *p* < 0.01, *** *p* < 0.001, **** *p* < 0.0001.

MHC Class I is a pre-requisite for responses to T cells including TIL ACT [26] and the loss of antigen presentation machinery is an important resistance mechanism of anti-PD1 therapy [27,28]. Inhibition of BRAF is associated with up-regulation of MHC Class I and presentation of antigen [29]. We therefore investigated if the addition of CDK4/6 inhibitor to BRAF-MEKi could further enhance MHC Class I expression in our models. Dabrafenib-trametinib led to a significant 2.4-fold upregulation of both H2Db and H2Kb over control in SM1WT1 (Figure 5A,B: *p* ≤ 0.01). Similarly, dabrafenib-trametinib upregulated both H2Db and H2Kb by 3.6-fold over DMSO in YOVAL1.1 (Figure 5D,E: *p* ≤ 0.05) and there was no significant difference with the addition of palbociclib in either cell line. Palbociclib itself did not alter YOVAL1.1 expression of MHC Class I (Figure 5F), while SM1WT1 H2Db and H2Kb expression increased by approximately 33% (Figure 5A,B). Representative FACS plots are shown in Figures S2 and S3.

Although BRAF-MEKi upregulates MHC Class I expression, it can also enhance PD-L1 expression thereby potentially counteracting immune responses. SM1WT1 PD-L1 expression was increased by 1.7-fold over DMSO control by both DT and DTP treatment (*p* < 0.002) (Figure 5C). YOVAL1.1 expression of PD-L1 was increased by 2.0-fold (*p* < 0.006) and 2.9-fold (*p* ≤ 0.016) with DT and DTP treatment, respectively (Figure 5F). Palbociclib itself did not alter expression of PD-L1 in either cell line (Figure 5C,F) indicating BRAF-MEKi was responsible for upregulation in the DTP treatment group.

In parallel with these experiments, we also assessed MHC Class I and PD-L1 expression in response to targeted therapy in the presence of IFN-γ. (Figure 5G–L). Under these conditions, DT and DTP treatment did not consistently increase H2Db and H2Kb expression compared to IFN-γ alone. Surprisingly, palbociclib treatment in the presence of IFN-γ reduced YOVAL1.1 H2Db expression (0.66-fold decrease, *p* < 0.0018) with a similar trend observed with H2Kb (0.674-fold decrease, *p* < 0.099). YOVAL1.1 PD-L1 expression was also inhibited with palbociclib plus IFN-γ treatment compared to IFN-γ control (0.474 decrease, *p* < 0.006). However, palbociclib did not alter MHC Class I expression in the human BRAF^V600^ mutant melanoma cell lines A375, HT144, SKMEL-28, and WM266-4 cell lines even in the presence of IFN-γ (Figure S4). Palbociclib enhanced PD-L1 expression in A375 in particularly with IFN-γ (Figure S5).

**Figure 6:**
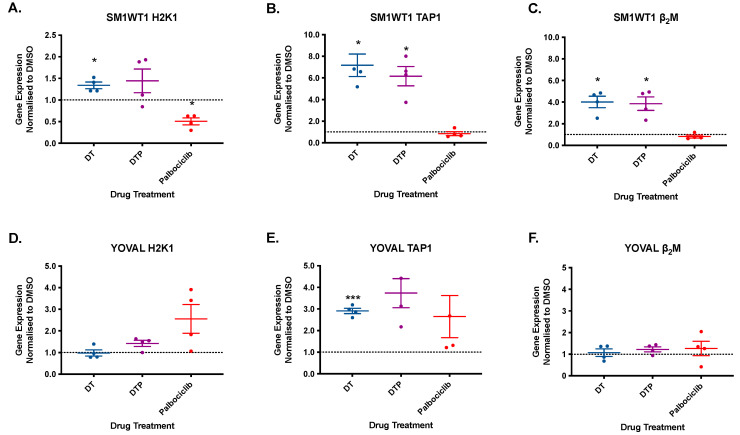
SM1WT1 and YOVAL1.1 gene expression of H2K1, TAP1 and β_2_M. SM1WT1 and YOVAL1.1 treated with control (DMSO), dabrafenib-trametinib (DT), dabrafenib-trametinib-palbociclib (DTP), and palbociclib monotherapy. Samples analyzed using real time PCR for H2K1 (**A**,**D**), TAP1 (**B**,**E**), and β_2_M (**C**,**F**). Mean ± SEM, *n* = 4. Gene expression normalized to DMSO control. Statistical significance was determined using one way ANOVA * *p* < 0.05, *** *p* < 0.001.

In order to explore the potential mechanism of MHC Class I upregulation induced by dabrafenib-trametinib ± palbociclib, real-time PCR of H2K1, Transport associated with Antigen Processing-1 (TAP1) and Beta-2-Microglobulin (β_2_M) was performed (Figure 6, Figure S6). Dabrafenib-trametinib ± palbociclib upregulated TAP1 (approx. 6–7-fold) and β_2_M (approx. 4-fold) mRNA expression in SM1WT1. H2K1 was also upregulated by dabrafenib-trametinib ± palbociclib although the increase observed by the drug triplet did not meet statistical significance. In contrast, palbociclib monotherapy down regulated H2K1 gene expression by 50% (*p* < 0.05: Figure 5A). In YOVAL1.1 cells, TAP1 appeared to be upregulated by dabrafenib-trametinib ± palbociclib although only the BRAF-MEKi group attained statistical significance (*p* < 0.001). Overall, there was no effect by BRAF-MEKi nor CDK4/6i on YOVAL1.1 H2K1 or β_2_M expression.

## 4. Discussion

The primary aim of this study was to assess whether the addition of palbociclib could augment anti-tumor responses in combination with adoptive cell transfer and BRAF-MEKi. Combination BRAF-MEKi can shape the tumor microenvironment by reducing immunosuppressive factors such as vascular endothelial growth factor, IL-6, IL-10, and TGF-β which corresponds with increasing tumor infiltrating lymphocytes [11,30]. Given the potential immunomodulation observed in previous studies, it is therefore logical to combine CDK4/6i with BRAF-MEKi. Previously we have shown that DTP elicited a robust anti-tumor response against YOVAL1.1 in vivo with overall survival of beyond 100 days compared to approximately 70 days for BRAF-MEKi [16]. Combination DTP increased T cell and CD8 infiltration in the tumor microenvironment while Treg populations were reduced compared to both controls and DT treated mice [16]. Critically DTP reduced CD103^+^ dendritic cell (DC) populations in the tumor microenvironment which accounted for resistance to combination anti-CTLA-4 and anti-PD1 antibodies after progression of the targeted therapy triplet [16]. Given this observation, we reasoned that DTP could also antagonize ACT, however in BRAFi sensitive YOVAL1.1 tumors combined DTP and OT-1 ACT further enhanced the durability and depth of response compared to either treatment alone.

CDK4/6 inhibitors have been shown to enhance the immunogenicity of cancer and modulate the tumor microenvironment. Goel et al. demonstrated CDK4/6i upregulated MHC Class I expression via upregulation of HLA-A, HLA-C, B_2_M and mRNA in breast cancer cell lines [17]. Similar upregulation of MHC Class I by CDK4/6i was also demonstrated in the murine melanoma cell line B16-OVA with co-culture experiments showing enhanced IFN-γ and TNF-α release when treated with OT-1 T cells [17]. Contrary to these prior studies, neither SM1WT1 nor YOVAL1.1 MHC Class I expression was altered by palbociclib monotherapy. BRAF-MEKi in both cell lines did enhance MHC Class I expression but there was no additional contribution with the addition of CDK4/6i. Furthermore, we did not observe changes of MHC Class I expression in several human melanoma cell lines which indicates the response to CDK4/6i is not universal.

CDK4/6i can also enhance inhibitory immune checkpoints namely PD-L1. Treatment with CDK4/6i inhibits speckle-type POZ protein (SPOP), a cullin E3 ubiquitin ligase protein that stimulates proteasome-mediated degradation of PD-L1 [20]. In this key paper, Zhang et al. demonstrated palbociclib upregulated PD-L1 in B16F10 in vivo. Additionally, they showed fluctuation of PD-L1 expression during cell cycle progression and palbociclib treatment consistently upregulated this immune checkpoint in a variety of different tissues such as brain, liver and colon. In our work we did not identify enhancement of PD-L1 expression in vitro with palbociclib or in combination with BRAF-MEKi in our melanoma models.

While we did not show palbociclib enhancement of either YOVAL1.1 or SM1WT1 MHC Class I expression, CDK4/6i itself can potentially enhance T cell and ACT activity. Palbociclib treatment is associated with upregulation of nuclear factor of activated T-cells (NFAT) in Jurkat cells with subsequent higher IL-2 and GM-CSF production [18]. Using a CRISPR/CAS9 screen on Jurkat T cells, retinoblastoma protein was also shown to be necessary for CDK4/6i upregulation of CD62L expression and skew TIL populations towards memory differentiation [21]. These studies also showed significantly enhanced CAR-T cell persistence and anti-tumor efficacy with palbociclib treatment indicating a potential role for CDK4/6i in ACT [21].

In the clinic, the targeted therapy triplet of BRAF-MEK-CDK4/6i could be employed in combination with TIL ACT. BRAFi have been combined with TIL ACT in small pilot studies with encouraging activity and safety [31,32,33]. In two trials, the BRAFi vemurafenib was commenced after excision of melanoma metastases for ACT production and briefly withheld during the pre-conditioning chemotherapy regimen for five days [32,33]. Objective responses were observed in 7 of 11 patients which included 2 participants with complete responses [33]. In a second trial, 6 of 16 patients (38%) exhibited objective responses at the 12 month mark with prolonged overall survival in responders varying from 38 to 66 months [32]. Importantly vemurafenib was able to prevent clinical progression during the three to six-week ACT manufacturing period which is a key limitation of this therapy. The BRAF-MEK-CDK4/6i combined with ACT might be of particular benefit for patients with poor prognostic features such as those with high volume disease or elevated lactate dehydrogenase (LDH) where both immune checkpoint inhibitors and combination BRAF-MEKi have substantially reduced efficacy [1,34]. Five-year progression free survival of patients with normal versus elevated LDH was 41% and 28%, respectively in patients treated with ipilimumab-nivolumab [1]. Moreover, only 8% of patients with elevated LDH treated with first line dabrafenib-trametinib had ongoing responses at the same time point indicating a critical need for new treatments in this poor prognostic group [2]. The rapid onset of responses with BRAF-MEKi and ACT might serve as useful treatments for these poor prognostic patients.

We acknowledge a limitation of this work is the use of murine melanoma rather than human ACT models. However, xenograft ACT models require immunodeficient mice which may confound outcomes. Furthermore, it would limit the ability to assess if depletion of tumor associated proinflammatory macrophages and cross-priming CD103^+^ dendritic cells which we have previously observed with BRAF-MEK-CDK4/6i treatment would limit the efficacy of concurrent ACT treatment [16]. Although CDK4/6i induces neutropaenia, which may bring safety concerns with pre-conditioning chemotherapy required for TIL ACT, the potential for CDK4/6i to modulate T cell populations to a memory phenotype might also be advantageous for ACT. Clinical data on the potential additive benefit of palbociclib with BRAF-MEKi is pending with the phase Ib CELEBRATE trial (NCT04720768) which investigates the addition of palbociclib to encorafenib-binimetinib. This eagerly awaited study might provide an additional targeted therapy regimen that could set the stage for future trials in combination with adoptive cell transfer.

## 5. Conclusions

Overall our findings indicate the BRAF-MEK-CDK4/6i can be employed in combination with ACT and provides additional evidence that supports clinical studies of CDK4/6i in combination with BRAF-MEKi in advanced melanoma. We did not find that CDK4/6i alone or in combination with BRAF-MEKi enhanced MHC Class I in YOVAL1.1 or SM1WT1. Consistent with other studies BRAF-MEK-CDK4/6i led to robust anti-tumor activity in both BRAFi resistant SM1WT1 and BRAFi sensitive YOVAL1.1 models and importantly did not adversely affect the response to ACT. This data supports the use of this novel triplet targeted therapy with adoptive cell transfer.

## Data Availability

The data is contained within the article or supplementary material.

## Supplementary Materials

The following are available online at https://www.mdpi.com/article/10.3390/cancers13246342/s1, Figure S1: FACS plots of pmel-1 adoptive cell transfer, Figure S2: Representative FACS plots of SM1WT1 treated with DMSO, Dabrafenib-Trametinib (DT), Dabrafenib-Trametinib-Palbociclib (DTP) and Palbociclib for 72 h, Figure S3: Representative FACS plots of YOVAL1.1 treated with DMSO, Dabrafenib-Trametinib (DT), Dabrafenib-Trametinib-Palbociclib (DTP) and Palbociclib for 72 h, Figure S4: Human melanoma cell lines and HLA-A, -B, -C expression with palbociclib, Figure S5: Human melanoma cell lines and PD-L1 expression with palbociclib, Figure S6: SM1WT1 and YOVAL1.1 gene expression of H2K1, TAP1 and β_2_M with Targeted Therapy IFN-γ Treatment.
