# Supplementary material for: Enhancing Adoptive Cell Transfer with Combination BRAF-MEK and CDK4/6 Inhibitors in Melanoma
Source: Cancers (Basel). 2021 Dec 17;13(24):6342. doi: 10.3390/cancers13246342 (PMC8699814; doi:10.3390/cancers13246342)
Supplement: Supplementary file 1 [file cancers-13-06342-s001.zip › cancers-1472547-supplementary.pdf]

cancers

Supplementary Materials: Enhancing Adoptive Cell Transfer with Combination BRAF-MEK and CDK4/6 Inhibitors in Melanoma

Peter Kar Han Lau, Carleen Cullinane, Susan Jackson, Rachael Walker, Lorey K. Smith, Alison Slater, Laura Kirby, Riyaben P. Patel, Bianca von Scheidt, Clare Y. Slaney, Grant A. McArthur and Karen E. Sheppard

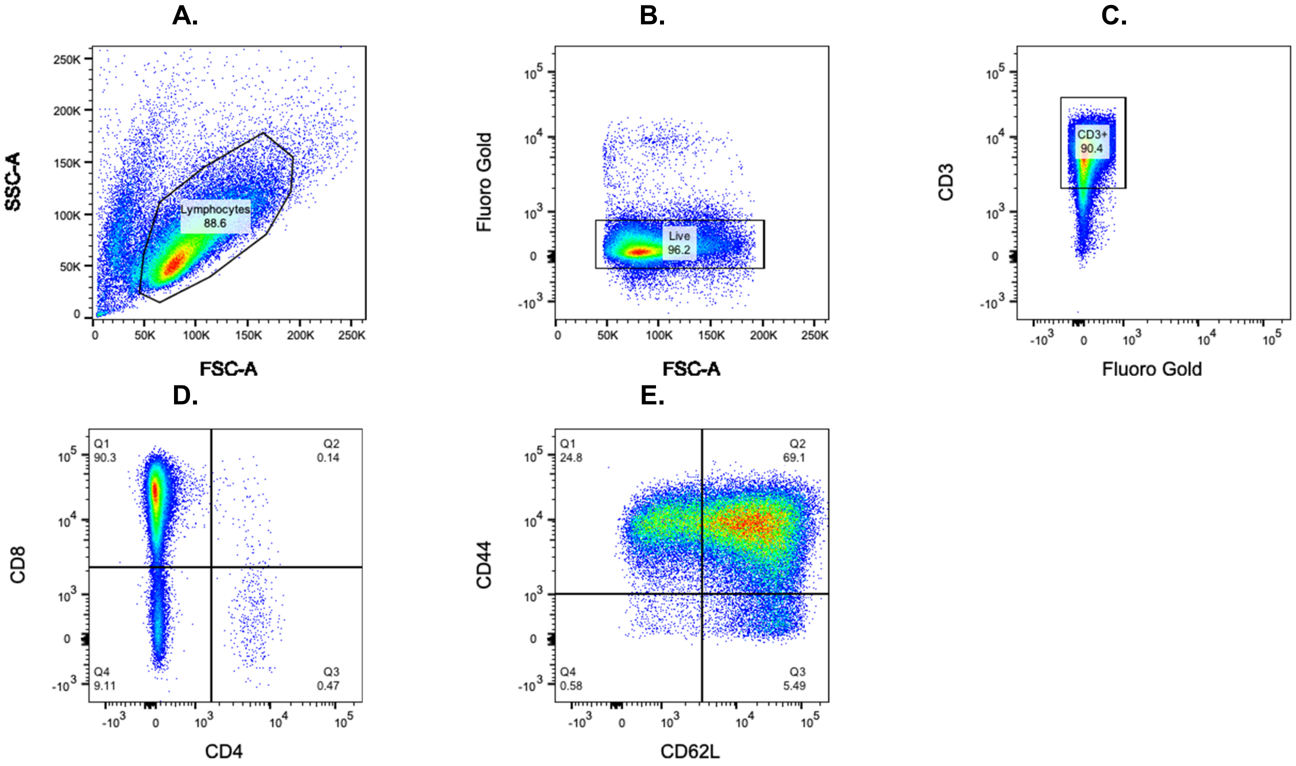

Figure S1. FACS plots of pmel-1 adoptive cell transfer. Forward and side scatter plots of pMel cells displayed in Panel **A**. Lymphocytes were gated using fluorogold as a viability marker (Panel **B**). Cells high in expression CD3 (Panel **C**) were predominantly CD8+CD4-ve cells (Panel **D**). This subpopulation displayed a predominant population of CD44^{hi} CD62L^{hi} phenotype displaying an activated T cell effector memory phenotype (Panel **E**).

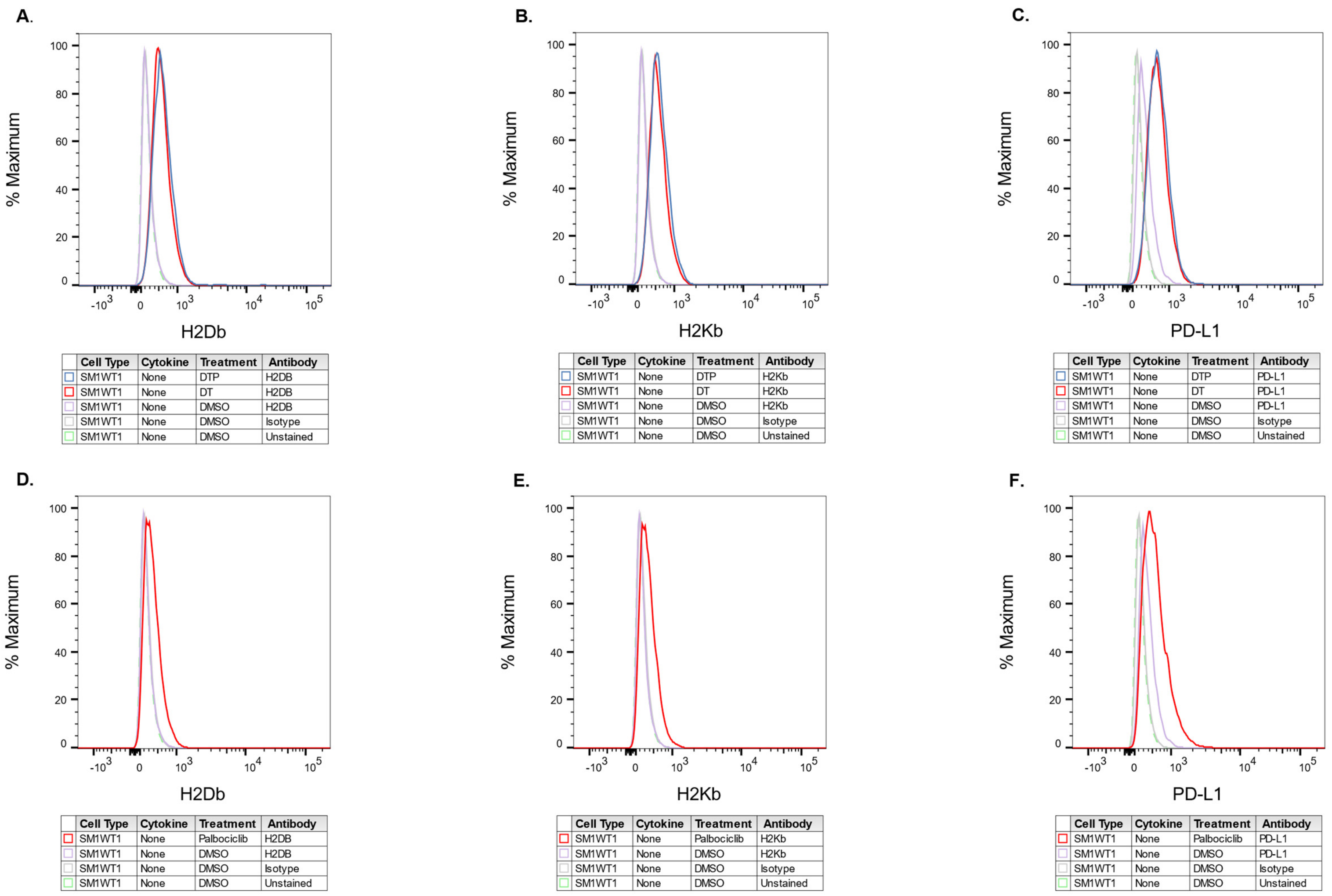

Figure S2. Representative FACS plots of SM1WT1 treated with DMSO, Dabrafenib-Trametinib (DT), Dabrafenib-Trametinib-Palbociclib (DTP) and Palbociclib for 72 hours. Panel A SM1WT1 H2Db FACS expression after 72 hour treatment with DMSO, DT or DTP. Panel B SM1WT1 H2Kb FACS expression after 72 hour treatment with DMSO, DT or DTP. Panel C SM1WT1 PD-L1 FACS expression after 72 hour treatment with DMSO, DT or DTP. Panel D SM1WT1 H2Db FACS expression after 72 hour treatment with DMSO or palbociclib. Panel E SM1WT1 H2Kb FACS expression after 72 hour treatment with DMSO or palbociclib. Panel F SM1WT1 PD-L1 FACS expression after 72 hour treatment with DMSO or palbociclib.

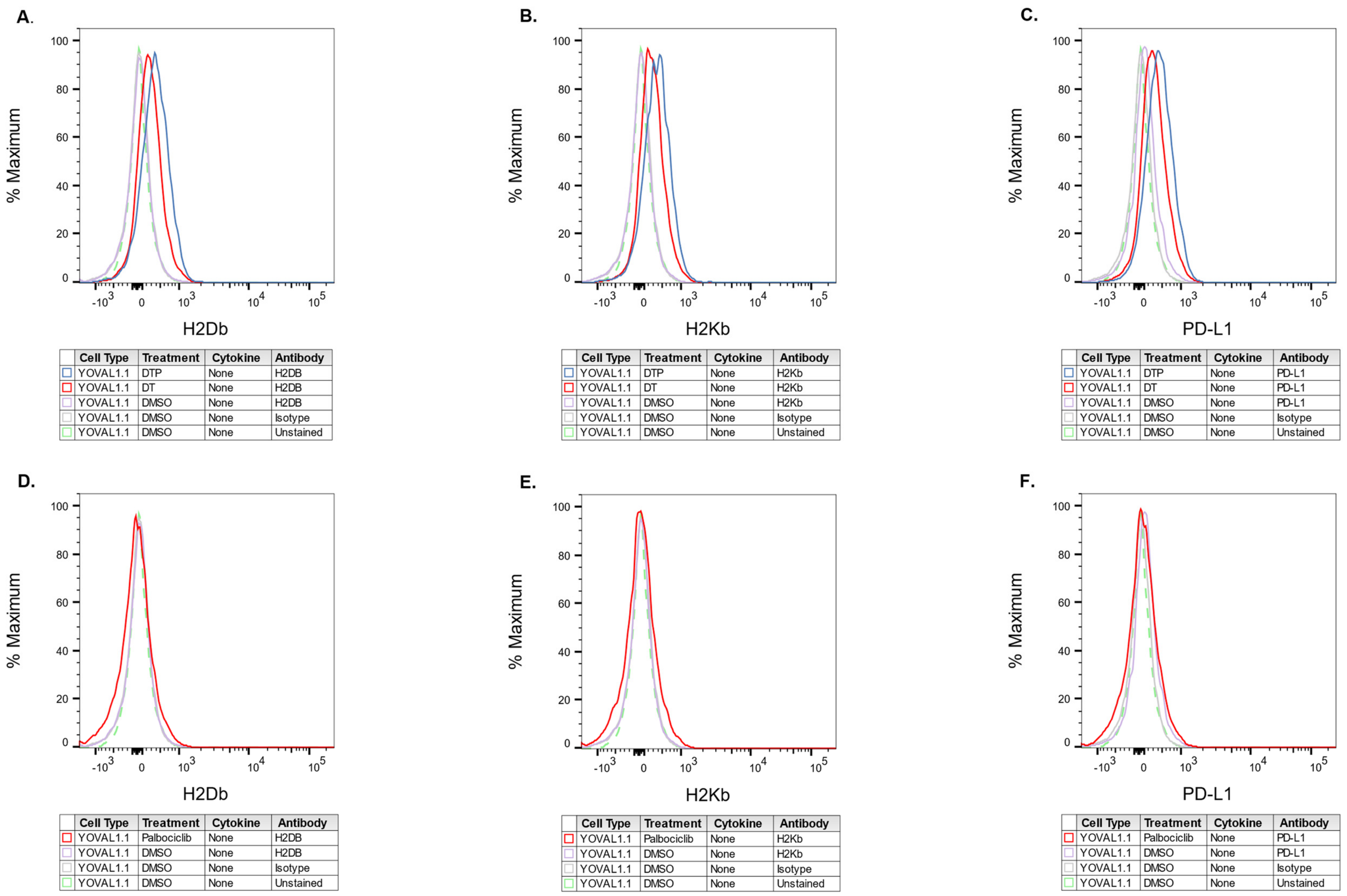

Figure S3. Representative FACS plots of YOVAL1.1 treated with DMSO, Dabrafenib-Trametinib (DT), Dabrafenib-Trametinib-Palbociclib (DTP) and Palbociclib for 72 hours. Panel A YOVAL1.1 H2Db FACS expression after 72 hour treatment with DMSO, DT or DTP. Panel B YOVAL1.1 H2Kb FACS expression after 72 hour treatment with DMSO, DT or DTP. Panel C YOVAL1.1 PD-L1 FACS expression after 72 hour treatment with DMSO, DT or DTP. Panel D YOVAL1.1 H2Db FACS expression after 72 hour treatment with DMSO or palbociclib. Panel E YOVAL1.1 H2Kb FACS expression after 72 hour treatment with DMSO or palbociclib. Panel F YOVAL1.1 PD-L1 FACS expression after 72 hour treatment with DMSO or palbociclib.

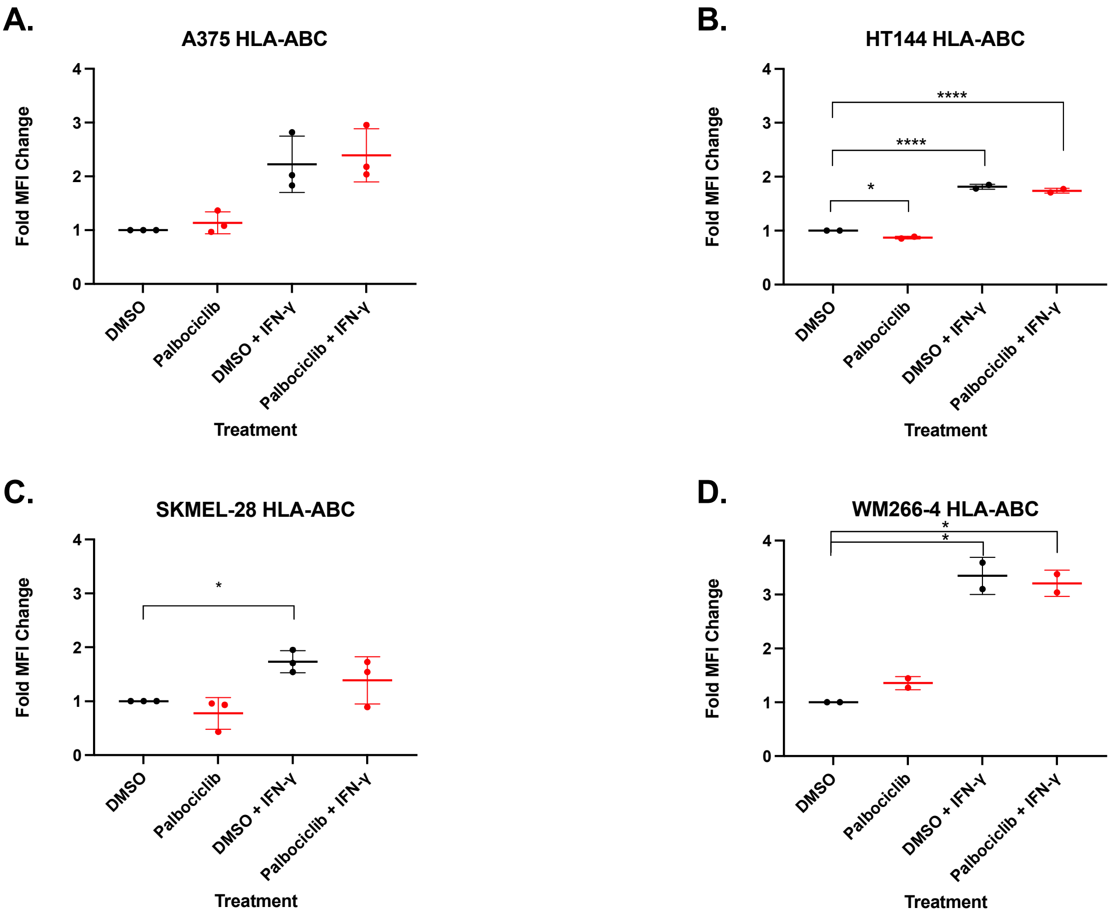

Figure S4. Human melanoma cell lines and HLA -A, -B, -C expression with palbociclib. Samples analysed using FACS for HLA -A, -B, -C. Median fluorescent intensity of the respective treatment group was normalised back to DMSO control and expressed as Fold MFI Change. A375 and SK-MEL-28 were treated with palbociclib (1 µM) ± IFN-γ (2 ng/ml). HT144 and WM266-4 were treated with palbociclib (500 nM) ± IFN-γ (2 ng/ml). All drug treatments were for 72 hours. Mean presented with error bars representing standard deviation. Statistical significance was determined using one way ANOVA with DMSO or DMSO + IFN- γ * $p < 0.05$, ** $p < 0.01$, *** $p < 0.001$, **** $p < 0.0001$.

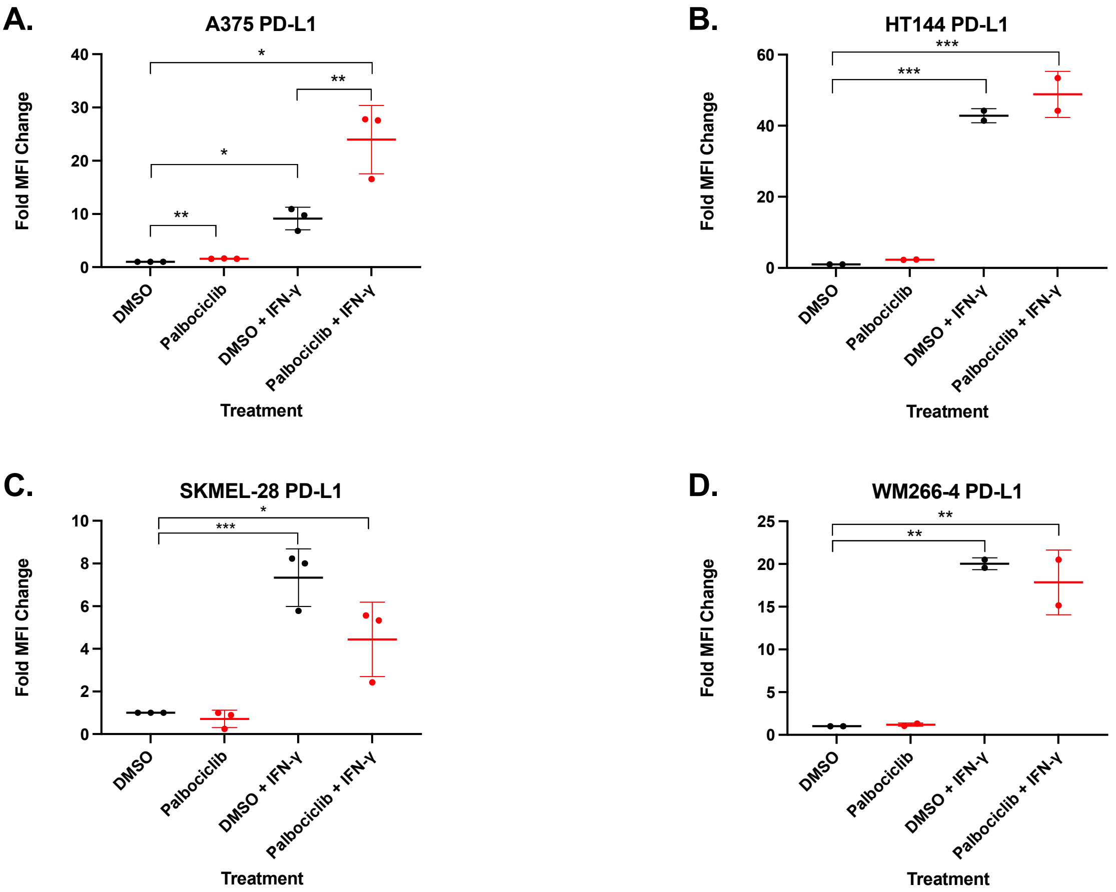

Figure S5. Human melanoma cell lines and PD-L1 expression with palbociclib. Samples analysed using FACS for PD-L1. Median fluorescent intensity of the respective treatment group was normalised back to DMSO control and expressed as Fold MFI Change. A375 and SK-MEL-28 were treated with palbociclib (1 µM) ± IFN-γ (2 ng/ml). HT144 and WM266-4 were treated with palbociclib (500 nM) ± IFN-γ (2 ng/ml). All drug treatments were for 72 hours. Mean presented with error bars representing standard deviation. Statistical significance was determined using one way ANOVA with DMSO or DMSO + IFN- γ * $p < 0.05$, ** $p < 0.01$, *** $p < 0.001$, **** $p < 0.0001$.

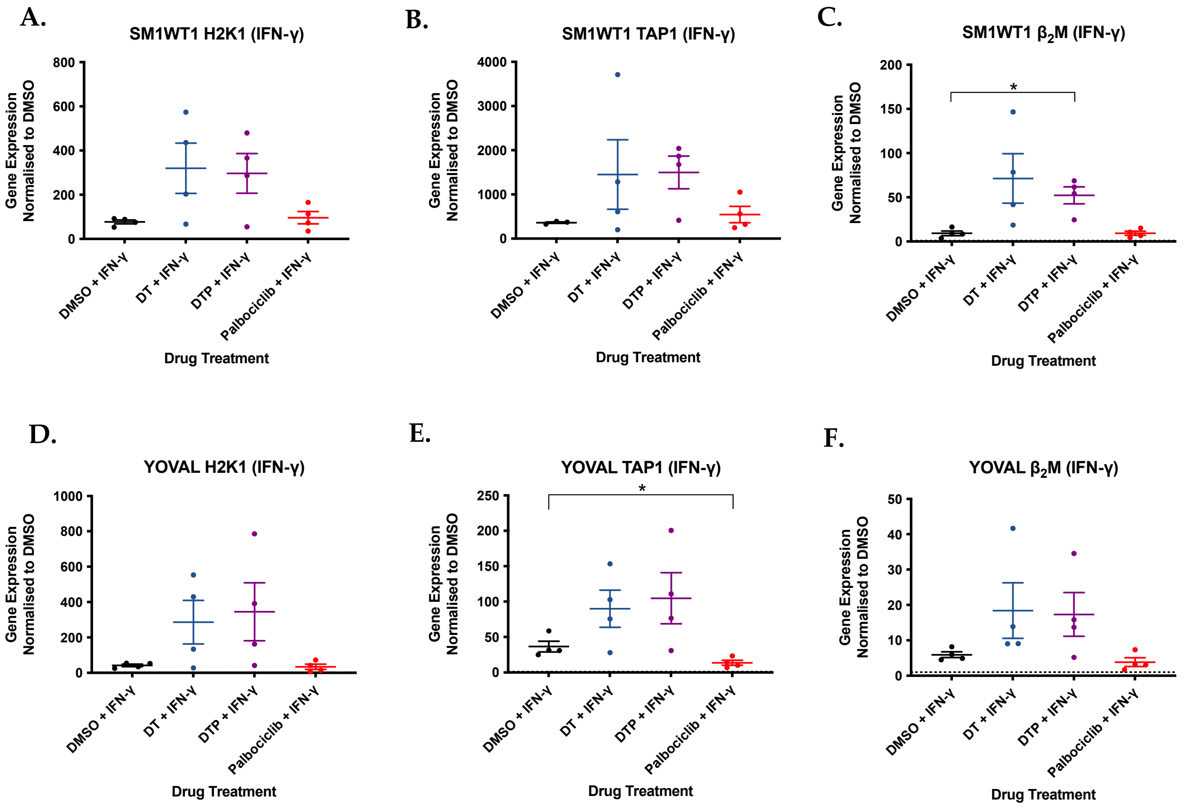

Figure S6. SM1WT1 and YOVAL1.1 gene expression of H2K1, TAP1 and β2M with Targeted Therapy IFN-γ Treatment. SM1WT1 and YOVAL1.1 treated with control (DMSO), dabrafenib-trametinib (DT), dabrafenib-trametinib-palbociclib (DTP) and palbociclib monotherapy with IFN-γ (2 ng/ml). Samples analyzed using real time PCR for H2K1 (**A**, **D**), TAP1 (**B**, **E**) and β2M (**C**, **F**). Mean ± SEM, *n*=4. Gene expression normalised to DMSO control. Statistical significance was determined using one way ANOVA * $p < 0.05$, ** $p < 0.01$, *** $p < 0.001$, **** $p < 0.0001$.
